# Circulating Levels of Ferritin, RDW, PTLs as Predictive Biomarkers of Postoperative Atrial Fibrillation Risk after Cardiac Surgery in Extracorporeal Circulation

**DOI:** 10.3390/ijms232314800

**Published:** 2022-11-26

**Authors:** Claudia Altieri, Calogera Pisano, Labriola Vincenzo, Maria Sabrina Ferrante, Valentina Pellerito, Paolo Nardi, Carlo Bassano, Dario Buioni, Ernesto Greco, Giovanni Ruvolo, Carmela Rita Balistreri

**Affiliations:** 1 Department of Cardiac Surgery, Tor Vergata University Rome, 00133 Rome, Italy; 2 Cellular and Molecular Laboratory, Department of Biomedicine, Neuroscience and Advanced Diagnostics (Bi.N.D.), University of Palermo, Corso Tukory 211, 90134 Palermo, Italy; 3 Cardiac Surgery Unit, Department of Clinical, Internal Medicine, Anesthesiology and Cardiovascular Sciences, Sapienza University Rome, 00161 Rome, Italy

**Keywords:** postoperative atrial fibrillation, cardiothoracic surgery, conventional extracorporeal circulation, serum ferritin levels, PW indices, POAF onset biomarkers

## Abstract

Postoperative atrial fibrillation (POAF) is the most common arrhythmia after cardiac surgery in conventional extracorporeal circulation (CECC), with an incidence of 15–50%. The POAF pathophysiology is not known, and no blood biomarkers exist. However, an association between increased ferritin levels and increased AF risk, has been demonstrated. Based on such evidence, here, we evaluated the effectiveness of ferritin and other haematological parameters as POAF risk biomarkers in patients subjected to cardiac surgery. We enrolled 105 patients (mean age = 70.1 ± 7.1 years; 70 men and 35 females) with diverse heart pathologies and who were subjected to cardiothoracic surgery. Their blood samples were collected and used to determine hematological parameters. Electrocardiographic and echocardiographic parameters were also evaluated. The data obtained demonstrated significantly higher levels of serum ferritin, red cell distribution width (RDW), and platelets (PLTs) in POAF patients. However, the serum ferritin resulted to be the independent factor associated with the onset POAF risk. Thus, we detected the ferritin cut-off value, which, when ≥148.5 ng/mL, identifies the subjects at the highest POAF risk, and with abnormal ECG atrial parameters, such as PW indices, and altered structural heart disease variables. Serum ferritin, RDW, and PTLs represent predictive biomarkers of POAF after cardiothoracic surgery in CECC; particularly, serum ferritin combined with anormal PW indices and structural heart disease variables can represent an optimal tool for predicting not only POAF, but also the eventual stroke onset.

## 1. Introduction

Postoperative atrial fibrillation (POAF) is a secondary form of AF from reversible cause [1]. POAF represents the most common arrhythmia after thoracic or cardiac surgery, having a prevalence of about 15% to 50% in patients subjected to coronary artery bypass surgery (CABG) [2], and of 60–80% in patients undergoing valve surgery [3,4]. POAF is associated with diverse severe complications, including stroke, cardiac failure, and cardiovascular death [3,4]. The risk of developing stroke, including stroke severity and mortality from stroke, is particularly frequent in post-cardiothoracic surgery AF patients [5,6]. All these complications need additional treatments, as well as prolonged hospital stays and increased costs [7]. A better understanding of the underlying mechanisms might enable the identification of both biomarkers and new therapeutic targets [1,3,4]. Thus, POAF in cardiac care remains a critical issue, although advances in cardiac surgery and perioperative treatment have been dramatically achieved [8,9,10,11]. Accordingly, the complete identification of the potential risk factors, mechanisms, and pathways involved in POAF onset, and especially in POAF after cardiothoracic surgery in CECC, constitutes a significant challenge, and might consequently enable the best patient outcomes after surgery to be achieved, such as less postoperative complications and long-term survival rates. Until now, POAF pathogenesis and pathophysiology have not been fully discovered [1,3,4], and this might be explained by the major clinical and research interest for AF, and not for POAF. Despite the existence of limited evidence in the literature, a multifactorial character for POAF onset and progression has been identified and suggests the surgery treatment as the first POAF risk factor, which induces hypercoagulation in the patients, with a high risk of bleeding. Another factor may occur after surgery, and determines sympathetic activation, causing heart rate elevation and catecholamine release, which leads the myocardium to develop arrhythmia. In addition, post-surgery electrolyte imbalances, transient hypoxemia, and electrophysiological disturbances have also been demonstrated to contribute to POAF onset. Furthermore, hypervolemia from intraoperative fluid administration seems also to induce right atrial stretching, causing an arrhythmia [1,3,4]. In addition to these mentioned risk factors, inflammation has been also reported as one of the major contributing pathophysiological factors in POAF occurrence. An increase in the circulating levels of elevated C-reactive protein (CRP) and interleukins, and a significative augment of leukocytes have been associated with the onset and progression of POAF, especially in post-cardiac surgery patients, even if the involved mechanisms are not well recognized [10,11,12,13]. Open heart surgery and CECC, however, contribute to evocating local and systemic inflammation. Systemic inflammation can cause diminished visceral blood flow resulting in mucosal ischemia, edema, and impaired mucosal transport [12,13,14,15,16,17,18]. Concerning local inflammation, research performed over the past 5 years evidence that atrial cardiomyocytes have diverse inflammatory signaling pathways that are demonstrated to be activated in both animal models and AF patients [12,13,14,15,16,17]. Such growing evidence on the crucial role of inflammation in POAF has also led to investigations on hematological indices as potential prognostic and predictive biomarkers for POAF. Hematological indices are obtained from complete blood count test, and comprise white blood cell (WBC), red cell distribution width (RDW), mean platelet volume (MPV), neutrophil/lymphocyte ratio (NLR), platelet/lymphocyte ratio (PLR), and the systemic immune-inflammation index (SII), a novel biomarker that develops on platelet count and NLR (SII = platelets count × NLR) [17,18]. Interesting associations have been demonstrated with POAF developed after CABG treatment [17,18]. Other blood factors have been evaluated in POAF patients as the parameters related to iron status detected at preoperative and postoperative times [19]. Among these, serum ferritin has been reported to be increased in AF and POAF patients. Precisely, a recent meta-analysis [20] conducted on 35,799 male and female individuals from the general Danish population demonstrated a significant association between the increased ferritin concentration and higher risk of AF in both sexes, even if the effect size appeared to be more pronounced in men. In addition, the authors of this study evidenced a growing enhancement in AF risk related to a stepwise increasing ferritin concentration, with a peak of the highest AF risk when the ferritin concentration is >600 µg/L in both men and women [20]. Sokal and coworkers [21] conducted research on 40 patients subjected to the first AF cryoablation procedure and followed for a period of follow-up time of 6 months. Increased levels of ferritin, combined with augmented concentrations of high sensibility-CRP, have been detected and suggested as optimal POAF biomarkers and useful in the evaluation of the efficacy of cryoablation. The observed rise in serum ferritin may be related to fact that such parameter represents the well-known biomarker of iron deficiency commonly present in patients with chronic inflammatory diseases. However, serum ferritin is also the typical biomarker of clinical inflammation and trauma used in the evaluation of acutely ill patients. In AF and POAF patients, ferritin synthesis correlates with the elevated proinflammatory cytokines production, the consequent increase in hepcidin, macrophages activation, and the sequestration of iron (functional iron deficiency). Iron uptake into macrophages, via transferrin or scavenger-receptor-mediated erythrophagocytosis, enhances intracellular iron levels and accelerates ferritin synthesis and release. On another hand, the growing enhancement of serum ferritin correlates with the grade of systemic and local inflammation, and consequently with a bad clinical status of patients [19]. 

Based on such evidence, we wanted to test for the first time the effectiveness of serum ferritin levels and the other hematological indices as POAF predictive risk biomarkers in 105 patients (mean age = 70.1 ± 7.1 years; 70 men and 35 females) subjected to cardiothoracic surgery and CECC. 

## 2. Materials and Methods

### 2.1. Study Population and Design

The study was started once approval was obtained from the local ethics committee. This was a retrospective observational cohort study conducted on consecutive patients, who experienced elective cardiothoracic surgery for coronaropathies (N = 22), valvopathies (mitral and/or aortic; N = 40), ascending aorta aneurysms (N = 37), or for a combined aorto-pathies (3 with valvopathies and ascending aorta aneurysms and 3 with coronaropathies and ascending aorta aneurysms) between January 2021 and March 2022, at the Cardiac surgery Unit at Tor Vergata University. Patients with a history of previous AF or other cardiac dysrhythmias, and those undergoing emergency and re-operative operations were excluded from the study. They had a mean age of 70.1 (±7.1 SD) years, constituting 70 men and 35 females. In addition, all patients were also divided into two groups, namely group 1 and group 0, according to the occurrence of POAF during the postoperative period (after 4 days) and the ferritin cut-off values. Patients’ preoperative baseline clinical characteristics, comorbid conditions, laboratory parameters, intraoperative data, postoperative outcomes, and complications were screened through the computerized medical database of the hospital, recorded, and compared between the groups (see Table 1, Table 2 and Table 3). Thus, the predictive risk factors and perioperative results of POAF after cardiothoracic surgery were determined.

### 2.2. Postoperative Monitoring

During the postoperative first 48 h in ICU, electrocardiogram (ECG), invasive central venous and arterial pressures, and oxygen saturation of the patients were continuously monitored, and arterial blood gas analyses were performed regularly every 2–4 h. Cardiac rhythms of patients were assessed by obtaining standard 12-lead ECGs every day for the remaining days until discharge. In addition, heart rate and rhythm were assessed by palpation of the radial pulse at least once every 4 h. An additional 12-lead ECG was obtained and analyzed in case of tachycardia, palpitation, or a suspicion of an irregular cardiac rhythm. POAF was diagnosed with the existence of an irregular RR interval and an absence of P wave on the ECG. Symptomatic or asymptomatic POAF usually develops between days 2 and 4 after surgery [1,10,11]. Thus, in this study, data on POAF incidence were assessed for the onset of POAF until patients were discharged from the hospital. 

### 2.3. Electrocardiographic and Echocardiographic Parameters in the Groups 0 and 1

Both groups (group 0 and 1, above mentioned) underwent electrocardiography (ECG) evaluations. P-wave indices dispersion (PWd), maximum and minimum P-wave (P-max and P-min) were assessed [22,23]. Measurement of P-wave parameters were obtained using calipers and magnifying lens, including five cardiac cycles in eight leads. Measurements were performed manually and by two physicians, who were blinded into the study. P wave duration (maximum and minimum) were measured in a 12-lead ECG. PWd was calculated as the difference between maximum and minimum P wave duration (PWd = P-max − P-min). The interatrial block (IAB) was defined as prolonged P wave duration (≥110 ms). Furthermore, 2D echocardiography analysis was also performed according to protocols previously described in the literature [24,25]. Briefly, 2D echocardiograms were performed using dedicated Philips iE33 Ultrasound systems with Vision 2011.

### 2.4. Laboratory Analysis

Blood samples were taken from a peripheral vein after a 6–8 h fasting period. The samples were placed into sterile tubes containing or not a standard amount of anticoagulant and were quickly delivered to the laboratory for the analysis. To determine preoperative values of the haemato-chemical parameters, the samples were studied in an automatic CBC analysis device (Beckman Coulter Inc., Brea, CA, USA). The studied and derived CBC parameters for this study were hemoglobin (HB), red cell distribution width (RDW), and platelet (PLT) counts, expressed among the patients as mean ± standard deviation. Furthermore, concentrations of ferritin (immunochemical method) and sideraemia (immunochemical method) were determined in standard way in the hospital laboratory using Roche Diagnostics reagents according to the manufacturer instructions.

### 2.5. Statistical Analysis

Normality distribution for quantitative variables was assessed by the Shapiro–Wilk. The Mann–Whitney U test was computed to compare continuous clinical variables expressed as mean ± standard deviation, while chi-squared tests or the Fisher’s exact test (count < 5) were used to compare categorical ones. Multiple explanatory variable logistic regression analysis was performed to determine the risk factors/covariates for POAF. Receiver-operating characteristic (ROC) curve analysis was performed to determine the cut-off values of selected variables (i.e., serum ferritin concentrations), via logistic regression, for POAF from area under the curve (AUC). The ROC curve analysis was performed using the “Optimal Cutpoints” library of R software. Pearson and Spearman linear correlation coefficient analyses were used to assess the relationship between ECG findings and serum ferritin levels. To estimate the odds of structural heart disease variables for abnormal PW indices, we performed a cross-sectional analysis after surgery on patients who underwent 2D echocardiograms. We utilized the logistic regression model to calculate odds ratios, adjusted for age, gender, COPD, previous CVD, and obesity. Data are presented as odds ratios (95% CI) for categorical variables and difference (95% CI) for continuous variables.

All statistical analyses were performed using R Statistical Software (version 3.5.3; R Foundation for Statistical Computing, Vienna, Austria). All tests were two-tailed, and a *p*-value ≤ 0.05 was considered indicative of a statistically significant association.

## 3. Results

### 3.1. Demographic and Basal Clinical Characteristics of Patients Enrolled in the Study

They are presented in Table 1. Precisely, we selected in the study 105 patients having a mean age of 70.1 (±7.1 SD) years, prevalently constituted by men for the 67%, and affected 83% by hypertension, 23% by diabetes, 34% by dyslipidemia, 22% by COPD, 30% by obesity, and with 30% having had previous cardiovascular events. The 30% and the 17% of the patients were, respectively subjected to ace inhibitors and amiodarone treatment. In addition, 53% of cases were smokers and the 21% ex-smokers. 

Of them, 35 cases (see Table 1) developed POAF within the 4 postoperative days. They were characterized by being older (mean age: 73.3 ± 7.6; *p* = 0.005 by *t* test) and predominately men (69%), and with the highest value of body surface area (BSA) and of percentages of obesity (57%), hypertension (91.4%), COPD (51.5%), and previous CVD (51.5%) (see Table 1).

### 3.2. Evaluation of Circulating Ferritin Levels and Other Haemato-Chemical Biomarkers in POAF vs. No POAF Cases

By comparing the serum levels of haematological indices in the patients with POAF vs. cases without POAF (see Table 2), we detected that POAF cases had significantly lower concentrations of HB and sideraemia than patients without POAF. In addition, POAF patients showed significantly higher concentrations and percentages of Ferritin and RDW, respectively (see Table 2). Furthermore, the circulating levels of PLT also resulted significantly higher in POAF cases. However, after adjustment for age, previous CVD, obesity, hypertension, COPD, and gender, we assessed significative differences persisting only between the concentrations of Ferritin and the POAF risk (*p* = 0.001, by a logistic regression analysis). POAF cases also developed postoperative complications (i.e., endotracheal intubation > 48 h, pericardial and pleural spillage, *p* = 0.049, by chi-squared test; data not shown) than cases without POAF and subjected to cardiothoracic surgery.

### 3.3. Cut-Off Values of Mean Serun Ferritin Levels for Predicting the Occurrence of POAF in the 105 Patients Enrolled

**Figure 1:**
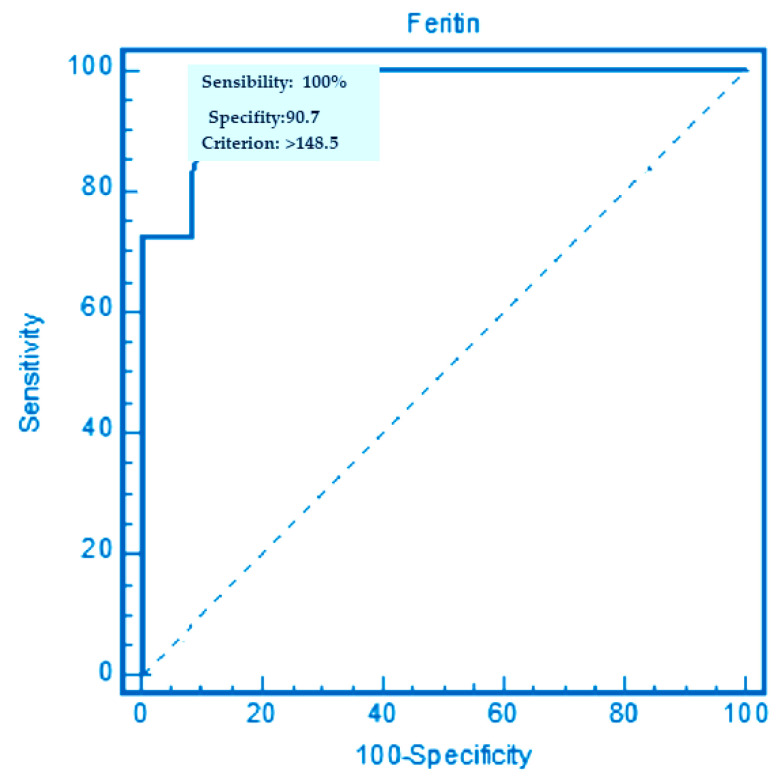
ROC curve, specificity, and sensibility representation for determining the cut-off value of serum ferritin to predict POAF onset.

**Figure 2:**
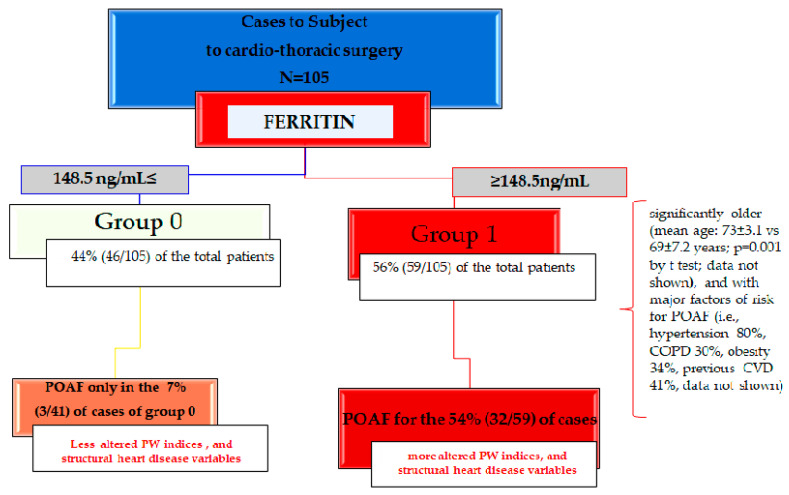
Flowchart identifying the two groups of cases, group 1 and 0, with serum ferritin levels equal to or higher than 148.5 ng/mL, which results in being predictive for developing POAF with a sensibility of 100% and specificity of 90.7% (see Figure 1).

The ROC curve analysis demonstrated that the value of 148.5 ng/mL constituted the cut-off value for predicting the occurrence of POAF with 100.0% sensitivity and 90.7% specificity (see Figure 1). The area under the ROC curve (AUC) was 0.987 (CI = 95%, range: 0.940–0.99; *p* = 0.0001) (see Figure 1). This value allowed us to identify two subgroups of patients from the 105 enrolled (see Figure 2), stratifying them by cut-off value: (a) group 1 with preoperative ferritin values ≥148.5 ng/mL, represented 56% (59/105) of the total patients, who developed, when subjected to cardiothoracic surgery, POAF in 54% (32/59) of cases; (b) group 0, having basal ferritin values ≤148.5 ng/mL, represented 44% of the total patients (46/105), who developed POAF in 7% (3/41) of cases. We illustrated the two groups using a flowchart’s representation (see Figure 2). Thus, the preoperative ferritin values ≥148.5 ng/mL are constant with identifying the cases enrolled with the highest risk of developing POAF. Accordingly, the group 0 with ferritin values ≤148.5 ng/mL resulted in 93% of cases not developing POAF after cardiothoracic surgery.

### 3.4. Major Prevalence of Risk POAF Factors in the Group 1 vs. Group 0

In addition, the cases of group 1 were significantly older (mean age: 73 ± 3.1 vs. 69 ± 7.2 years; *p* = 0.001 by *t* test; data not shown), and with major risk factors for POAF (i.e., hypertension 80%, COPD 30%, obesity 34%, previous CVD 41%, data not shown), than the cases of group 0, even if no significant differences were detected by comparing the values. In addition, they demonstrated significantly lower levels of HB and sidereamia than the subjects of group 0, and significant higher levels of PTLs and percentages of RDW, as shown in Table 3.

### 3.5. Significant Correlation between PWd, P-min, and P-max with Mean Serum Ferritin Levels and the Percentage of RDW in the Case Group 1 vs. Group 0

By using Pearson correlation test, we detected a statistically significant correlation between PWd, P-min, and P-max with mean serum ferritin levels and the percentage of RDW in the case group 1. In the cases of group 0, there was only a weak correlation between mean serum ferritin levels and PWd (see Table 4). 

### 3.6. The Association of PW Indices with Structural Heart Disease Variables in the Group 1 after Surgery

The association of PW indices with structural heart variables is displayed in Table 5. After adjustment for age, gender, COPD, previous CVD, and obesity, all PW indices were independently associated with left atrial enlargement, greater left ventricular mass, and greater left ventricular end diastolic dimension. In addition, they were associated with lower left ventricular ejection fraction, and PWd and Pmax were associated with lower left atrial emptying fraction, and PWd was also associated with lower left atrial global longitudinal strain.

## 4. Discussion 

The data obtained demonstrated that POAF patients showed significantly higher concentrations, absolute values, and percentages, of ferritin, RDW, and PLTs, respectively. However, after adjustment for other risk POAF variables, the ferritin resulted to be the independent factor associated with the onset POAF risk. Such an interesting result led us to detect the ferritin cut-off value, which, when equal to or greater than the value of 148.5 ng/mL, identifies the subjects at the highest risk of developing POAF. The high-risk POAF group was defined by us as group 1, differentiating it from group 0 who had values of ferritin ≤148.5 ng/mL. The cases of group 1 were significantly older (mean age: 73 ± 3.1 vs. 69 ± 7.2 years; *p* = 0.001 by *t* test; data not shown), and with major factors of risk for POAF (i.e., hypertension 80%, COPD 30%, obesity 34%, previous CVD 41%, data not shown), than the cases of group 0, even if no significant differences were detected by comparing the values. In addition, they showed to have significant lower levels of HB and sidereamia than the subjects of group 0, and significant higher levels of PTLs and percentages of RDW. We also found that in the cases of group 1, the serum ferritin correlated with more altered PW indices than the cases of group 0. Precisely, the PW indices were significantly prolonged in the patients of group 1 compared to cases of group 0 and resultingly were significantly associated with structural heart disease variables, after adjustment for risk POAF factors, such as age, gender, COPD, previous CVD, and obesity. Thus, this likely suggests that the combination of serum ferritin values with PW indices could be a strong biomarkers tool for predicting not only POAF, but also an eventual stroke, in the primary care setting of cases subjected to cardiothoracic surgery. 

These findings demonstrated, for the first time, the importance of detecting serum ferritin values, and if ≥148.5 ng/mL, in our case in the individuals subject to cardiothoracic surgery, this suggests the high probability of developing POAF [26,27] after the surgery treatment in CECC. This evaluation is always underestimated, as well as the screening of total iron parameters. They can evidence not only an iron deficiency status accompanied by clinical symptoms (i.e., fatigue, which is not specific and often confused with the symptoms of primary diseases), but they can also provide information about the clinical status of a patient related to the degree of systemic inflammation. It is well recognized, indeed, that the status of iron deficiency is recurrent in patients with chronic inflammatory diseases (such as chronic cardiovascular diseases [12,13,14,15,16,17], i.e., coronaropathies, valvopathies, and ascending aorta aneurysms, affecting the patients of our study), which significantly correlates with their severity. Iron deficiency is mediated by high systemic levels of cytokines, which evocate a parallel increase in hepcidin concentrations. In turn, hepcidin causes the sequestration of iron in cells of the reticuloendothelial system, determining the onset of a functional iron deficiency, even without anemia. The development of iron deficiency, even if functional, exacerbates underlying chronic diseases, alters erythropoiesis, and represents an independent factor of morbidity and mortality. In daily practice, the the iron body status is evaluated by detecting ferritin concentrations, because it reflects the iron body storage and transferrin saturation, which reproduces the transport of iron [28,29,30]. The serum concentration of ferritin is usually augmented in cases with a systemic inflammatory body condition likely related to diverse chronic inflammatory diseases, even if there is still not a scientific and clinical consensus on a threshold value to be used in chronic inflammatory conditions [28,29,30]. However, international guidelines define the iron deficiency condition as when serum ferritin values are <100 μg/L, either related or not to a transferrin saturation <20% and suggest that attention should be paid to the condition of iron depletion. In addition, it has been reported that high serum ferritin levels correlate with the aging process [28,29,30]. Accordingly, we detected that the patients of group 1 were older and with a worse clinical history than the patients of group 0, and with the highest levels of ferritin, which suggested in the complex a severer inflammatory status and clinical conditions than the components of group 0. They also had lower levels of HB and higher percentages and levels of RDW and PTLs, as mentioned above, which evidenced a severity grade of the inflammatory status and clinical conditions. Such erythropoiesis disturbances have been demonstrated to be likely determined by the iron sequestration in the macrophages. Two recent studies [31,32] have, indeed, demonstrated significant associations between RWD, the sensitive marker of increased red cells turnover [33,34], and AF in general populations. In addition, Topal and coworkers [17] have recently proved that the median values of white blood cell, platelet, neutrophil, neutrophil/lymphocyte ratio, and platelet/lymphocyte ratio, were significantly greater in cases with AF and that were undergoing off-pump coronary artery bypass grafting than in those cases in the non-AF group. Likewise, Sayin and coworkers [35] have also documented that PLT counts accompanied by the evaluation of other immune-inflammatory indexes can predict new onset AF in cases affected by acute coronary syndrome. Guzelburc and colleagues [36] have also detected a significant relationship between preoperatively calculated Platelet max index (PMI) and POAF, concluding that PMI may be used to predict patients at high risk of developing POAF. Until now, there have been no studies in the literature on the significant relationship between preoperatively calculated ferritin concentration and POAF. Of the limited number of studies, represented by precisely two works, one examined one the association with AF only in the general population, and the other in AF after cryoablation. Of note is, however, the first work, a meta-analysis conducted on 35,799 male and female individuals from the general Danish population [20], while the study from Sokal and coworkers [21] was conducted on 40 patients subjected to the first AF cryoablation procedure. Thus, there is the necessity to validate our data in a larger number of cases and to perform prospective studies rather than retrospective investigations—our study suffers from this limitation, as well as a relatively reduced number of cases—to evaluate other immune and inflammatory parameters demonstrated to be associated with POAF in other recent studies. 

## 5. Conclusions 

**Figure 3:**
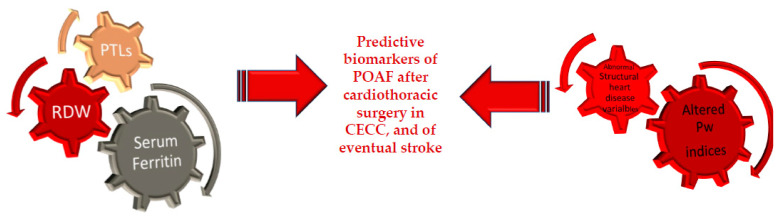
Serum ferritin, RDW and PLTs combined with anormal PW indices and structural heart disease variables constitute a strong toll for predicting POAF and an eventual stroke onset.

Our study, to our knowledge, represents the first study to stress serum ferritin, RDW, and PTLs as predictive biomarkers of POAF after cardiothoracic surgery in CECC. In addition, our study evaluates for the first time the power of serum ferritin in combination with anormal PW indices and structural heart disease variables for constituting a strong toll for predicting an eventual stroke onset (see Figure 3). Until now, ECG atrial parameter alterations have represented the approach to diagnosing AF. However, the new guidelines, published in 2020, focus their attention on AF by underlying that such disease requires a multifaceted, holistic, and multidisciplinary management approach, with the active involvement in partnership with clinicians, including cardiologists with varying subspecialty expertise, cardiac surgeons, methodologists, and clinical pathologists [37]. Nevertheless, they do not mention POAF or the iron parameters for evaluating at-risk AF patients, but they stress of use the CHA2DS2-VASc score (Congestive heart failure, Hypertension, Age ≥75 years, Diabetes mellitus, Stroke, Vascular disease, Age 65–74 years, Sex category (female)) in their diagnosis work-up and follow-up combined with the kidney biomarkers and full cell blood count [36]. In contrast, a recent work suggests the relevance of refining the biomarkers, the CHA2DS2-VASc score for predicting POAF and stroke [38]. It precisely reports that combining CHA2DS2-VASc score with EGC parameters, and particularly with PW indices, is possible to obtain a better tool for predicting AF and its major complication, such as stroke [38]. The CHA2DS2-VASc score has been shown to have predictive value specifically in the absence of detected AF. Of consequence, it is necessary to know if patients without pre-surgery AF would benefit from anticoagulation treatment guided by the CHA2DS2-VASc score or not. Measurement of prothrombotic atrial remodeling may suggest, particularly in patients without pre-surgery AF, the benefit of anticoagulant treatments, because in such patients a cardioembolic stroke might develop. PW indices, therefore, represent an optimal tool in stroke-risk prediction and consent to guide the anticoagulation therapy in people without AF in addition to enhancing stroke risk prediction in people with AF.

**Figure 4:**
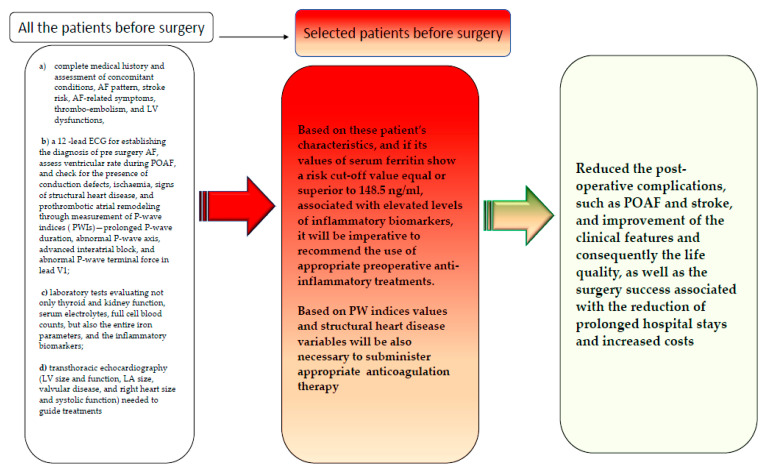
Our proposed clinical assessment to apply to all the patients to undergo cardiothoracic surgery in CECC, and particularly those with cardiovascular risk factors/comorbidities, and recommendations.

Based on the data obtained in our study, we emphasize that all the patients undergoing cardiothoracic surgery in CECC, and particularly those with cardiovascular risk factors/comorbidities, should benefit from a complete clinical assessment including: (a) the complete medical history and assessment of concomitant conditions, AF pattern, stroke risk, AF-related symptoms, thrombo-embolism, and LV dysfunctions; (b) a 12 -lead ECG for establishing the diagnosis of pre surgery AF, assess ventricular rate during POAF, and check for the presence of conduction defects, ischaemia, signs of structural heart disease, and prothrombotic atrial remodeling through measurement of P-wave indices (PWIs)—prolonged P-wave duration, abnormal P-wave axis, advanced interatrial block, and abnormal P-wave terminal force in lead V1; (c) laboratory tests evaluating not only thyroid and kidney function, serum electrolytes, and full cell blood counts, but also the entire iron parameters and the inflammatory biomarkers; (d) transthoracic echocardiography (LV size and function, LA size, valvular disease, and right heart size and systolic function) is needed to guide treatments. Based on these patients’ characteristics, and if their values of serum ferritin show a risk cut-off value associated with elevated levels of inflammatory biomarkers—reported in our study to be equal or superior to 148.5 ng/mL—it will be imperative to recommend the use of appropriate preoperative anti-inflammatory treatments for reducing post-operative complications, such as POAF and stroke, and improving clinical features and consequently life quality, as well as the surgery success associated with the reduction in prolonged hospital stays and increased costs (see Figure 4). Certainly, our study needs to be validated with other works, and possibly multicentered and larger populations, and not retrospective, to suggest the recommendations illustrated in Figure 4 and for them to be included in precise POAF guidelines.

## Figures and Tables

**Table 1:** Demographic and basal clinical characteristics of patients.

Variables	Overall	Without POAF	With POAF	*p*-Value
	N = 105	N = 70 (67.0)	N = 35 (33.0)	
Gender, n (%), n (%):				
F	35 (33.0)	21 (30.0)	11 (31.0)	0.79 ^†^
M	70 (67.0)	49 (70.0)	24 (69.0)	
Age (mean ± SD)	**70.1 ± 7.1**	**67 ± 6.8**	**73.3 ± 7.6**	**0.005 ^**
Body surface area (BSA), (mean ± SD)	1.81 ± 4.8	1.82 ± 4.6	1.92 ± 4.8	0.44 ^
HYPERTENSION, n (%), n (%):				0.06 ^§^
No	18 (17.0)	21(30.0)	3 (9.6)	
YES	87 (83.0)	49 (70.0)	32 (91.4)	
Smoking, n (%):				0.84 ^†^
Ex	22 (21.0)	14 (20.0)	8 (23.0)	
No	27 (26.0)	18 (26.0)	9 (26.0)	
YES	56 (53.0)	38 (54.0)	18 (51.0)	
DIABETES, n (%):				0.45 ^§^
No	82 (78.0)	55 (78.0)	27 (77.0)	
YES	23 (22.0)	15 (22.0)	8 (23.0)	
DYSLIPIDEMIA, n (%):				0.12 ^§^
No	73 (69.0)	50 (71.0)	23 (66.0)	
YES	32 (31.0)	20 (29.0)	12 (34.0)	
Chronic obstructive pulmonary disease (COPD), n (%):				**0.001** ^§^
No	80 (78.0)	**63 (90.0)**	**17 (48.5)**	
YES	25 (22.0)	**7 (10.0)**	**18 (51.5)**	
OBESITY, n (%):				**0.003** ^§^
No	73 (70.0)	**58 (83.0)**	**15 (43.0)**	
YES	32 (30.0)	**12 (17.0)**	**20 (57.0)**	
Previous cardiovascular diseases (CVD), n (%):				**0.003** ^§^
No	73 (70.0)	**56 (80.0)**	**17 (48.5)**	
YES	32 (30.0)	**14 (20.0)**	**18 (51.5)**	
Amiodarone., n (%):				0.29 ^§^
No	87 (83.0)	61 (87.0)	26 (74.0)	
YES	18 (17.0)	9 (13.0)	9 (26.0)	
ACE-INHIBITORS., n (%):				0.497 ^†^
No	73 (70.0)	45 (64.0)	28 (80.0)	
YES	32 (30.0)	25 (36.0)	7 (20.0)	

^§^*p*-value derived from Fisher exact test; ^†^*p*-value derived from the Chi-squared test; ^ *p*-value derived from *t* test.

**Table 2:** Preoperative circulating levels of haemato-chemical biomarkers in the patients enrolled in the study.

Variables	Overall	Without POAF	With POAF	*p*-Value ^ *(POAF Cases* vs. *Cases without POAF)*
	N = 105	N = 70	N = 35	
Hemoglobin, HB (g/dL) (mean ± SD)	13.1 ± 4.2	**12.8 ± 5.6**	**10.4 ± 3.6**	**0.024**
PLT 10^3^/µL	227.3 ± 1058	**230 ± 5.7**	**251 ± 5.6**	**<0.0001**
CREATININE (mg/dL)	1.30 ± 7.2	0.97 ± 7.2	1.48 ± 8.2	0.36
International normalized ratio (INR)	1.12 ± 0.8	1.12 ± 2.9	1.21 ± 5.5	0.42
Mg (mg/dL)	2.11 ± 2.4	1.92 ± 1.9	1.9 ± 1.9	0.40
K (mEq/L)	4.28 ± 2.5	4.20 ± 2.5	4.18 ± 2.8	0.41
RDW (%)	13.9. ± 3.1	**12.5 ± 3.5**	**16 ± 4.1**	**0.0003**
FERRITIN (ng/mL)	216.2 ± 3.5	**232.5 ± 6.2**	**286 ± 2.7**	**<0.0001**
SIDERAEMIA	82 ± 2.8	81 ± 3.2	75 ± 4.6	**0.04**

^ *p* values detected by *t* test.

**Table 3:** Hematological biomarkers, and demographic and clinical features in the two subgroups obtained by stratifying for the ferritin value of 148.5 ng/mL detected by tree test analysis.

Variables	Group 0	Group 1	*p*-Value ^
Hemoglobin, HB (g/dL) (mean ± SD)	**13.5 ± 4.1**	**11.5 ± 2.6**	**0.01**
PLT 10^3^/µL	**20 ± 4.9**	**24 ± 6.1**	**<0.0001**
CREATININE (mg/dL)	1.02 ± 5.6	1.14 ± 5.6	0.49
International normalized ratio (INR)	1.03 ± 1.5	1.11 ± 3.2	0.48
Mg (mg/dL)	1.83 ± 1.8	1.8 ± 4.1	0.48
K (mEq/L)	4.10 ± 1.5	4.08 ± 2.4	0.49
RDW (%)	**13.2 ± 3.1**	**16.0 ± 4.2**	**0.04**
SIDERAEMIA	86 ± 6.1	80 ± 3.1	**<0.0001**

^ *p* values detected by *t* test.

**Table 4:** Correlation between PWd, P-min, P-max, and mean serum ferritin levels, and the percentage of RDW in the case Group 1 vs. Group 0.

Variables	Group 1			Group 0		
Mean serum ferritin	PWd	P-max	P-min	Pwd	P-max	P-min
Pearson correlation	0.89	0.88	0.83	0.66	0.41	0.38
*p* value	0.0001	0.0001	0.0001	0.046	0.059	0.061
RDW (%)						
Pearson correlation	0.79	0.77	0.71	0.51	0.40	0.37
*p* value	0.0002	0.0002	0.0002	0.06	0.06	0.07

By Pearson correlation test; PWd: P-wave dispersion, P-Max: P maximum, P-Min: P minimum.

**Table 5:** Association Between Structural Heart Disease and P-Wave Indices in the Group 1.

Structural Heart Disease Variables *				
Left atrial volume index >34 mL/m^2^	PWd 1.49 (1.36–1.76) ^§^	P-max 1.45 (1.29–1.74) ^§^	P-min 1.29 (1.29–1.74) ^§^	IAB 1.29 (1.29–1.74) ^§^
Left ventricular mass index >115 g/m^2^ for men, >95 g/m^2^ for women	1.61 (1.42–1.88) ^§^	1.09 (1.01–1.41) ^‡^	1.06 (1.74–2.43) ^‡^	1.02 (1.01–1.14) ^‡^
Left ventricular end diastolic diameter >5.8 cm for men, >5.2 cm for women	2.25 (1.65–3.22) ^§^	1.52 (1.24–2.18) ^§^	1.39 (1.24–2.08) ^§^	1.19 (1.01–1.64) ^§^
Left ventricular ejection fraction <52% for men, <54% for female	1.36 (1.16–1.69) ^§^	1.18 (1.05–1.27) ^‡^	1.12(1.10–1.34) ^‡^	0.29 (1.29–1.74) ^§^
LAGLS %, difference (95% CI)	−1.59 (−2.33, −0.77) ^§^	−1.19 (−1.86, −0.33)^§^	−1.10 (−1.86, −0.33) ^§^	−1.29 (1.29–1.74) ^§^
LAEF %, difference (95% CI)	−2.79 (−3.29, −1.60) ^§^	−1.17 (−3.01, −0.30) ^‡^	−0.06 (−1.48, 1.32)	−1.97 (−4.85, 2.01)

IAB indicates interatrial block; LAEF, left atrial emptying fraction; LAGLS, left atrial global longitudinal strain; PWd: P-wave dispersion, P-Max: P maximum, P-Min: P minimum; * Data displayed as odds ratio (95% CI) unless otherwise stated. Difference is the difference in number of units (% for LAGLS and LAEF) between the cases of group 1 with abnormal PW indices and cases of group 1 with normal PW indices; † Logistic regression model was used adjusted for age, gender, COPD, previous CVD, and obesity. ^‡^ *p* < 0.05; ^§^ *p* < 0.01.

## Data Availability

Not applicable.
